# Outcomes of Optimized over Standard Protocol of Rabbit Antithymocyte Globulin for Severe Aplastic Anemia: A Single-Center Experience

**DOI:** 10.1371/journal.pone.0056648

**Published:** 2013-03-15

**Authors:** Xingxin Li, Jun Shi, Meili Ge, Yingqi Shao, Jinbo Huang, Zhendong Huang, Jing Zhang, Neng Nie, Yizhou Zheng

**Affiliations:** Severe Aplastic Anemia Studying Program, State Key Laboratory of Experimental Hematology, Institute of Hematology & Blood Diseases Hospital, Chinese Academy of Medical Science & Peking Union Medical College, Tianjin, P.R. China; University of Leicester, United Kingdom

## Abstract

**Background:**

Previous reports showed that outcome of rabbit antithymocyte globulin (rATG) was not satisfactory as the first-line therapy for severe aplastic anemia (SAA). We explored a modifying schedule of administration of rATG.

**Design and Methods:**

Outcomes of a cohort of 175 SAA patients, including 51 patients administered with standard protocol (3.55 mg/kg/d for 5 days) and 124 cases with optimized protocol (1.97 mg/kg/d for 9 days) of rATG plus cyclosporine (CSA), were analyzed retrospectively.

**Results:**

Of all 175 patients, response rates at 3 and 6 months were 36.6% and 56.0%, respectively. 51 cases received standard protocol had poor responses at 3 (25.5%) and 6 months (41.2%). However, 124 patients received optimized protocol had better responses at 3 (41.1%, *P* = 0.14) and 6 (62.1%, *P* = 0.01). Higher incidences of infection (57.1% *versus* 37.9%, *P* = 0.02) and early mortality (17.9% *versus* 0.8%, *P*<0.001) occurred in patients received standard protocol compared with optimized protocol. The 5-year overall survival in favor of the optimized over standard rATG protocol (76.0% *versus*. 50.3%, *P*<0.001) was observed. By multivariate analysis, optimized protocol (RR = 2.21, *P* = 0.04), response at 3 months (RR = 10.31, *P* = 0.03) and shorter interval (<23 days) between diagnosis and initial dose of rATG (RR = 5.35, *P* = 0.002) were independent favorable predictors of overall survival.

**Conclusions:**

Optimized instead of standard rATG protocol in combination with CSA remained efficacious as a first-line immunosuppressive regimen for SAA.

## Introduction

The worse prognosis of severe aplastic anemia (SAA) patients were not improved until the introduction of bone marrow transplant (BMT) and immunosuppressive therapy (IST) by antithymocyte globulin (ATG) [1], [2], [3], [4]. At the beginning of 1980s, Gluckman et al [5] and Champlin et al [6] reported the efficacy of ATG for SAA patients ineligible for BMT, and response rates using ATG alone ranged between 30% to 70% in larger series reports, which also pointed out the risk of late therapy failure due to relapse [7], [8]. Thus, several immunosuppressive agents in combination with ATG were explored to improve hematological response, sustain such response for longer period, and prevent subsequent relapse. In the 1990s, the combination of cyclosporine (CSA) with ATG was proven effective in increasing the response rate and survival [9], [10], which has been considered standard first-line IST for SAA with a 60–70% response rate and 70–80% long-term survival probability [11], [12], [13], [14].

But various ATG preparations with different stimulating antigens, animal sources, and dose of administration have exerted different immunosuppressive effects on T lymphocytes, showed variable response rates in SAA [14]. Outcomes of six large randomized clinical trials have documented the efficacy of horse ATG (hATG), including ATGAM® (Pfizer, New York, NY) in the United States [10], [15] and Lymphoglobulin® (Genzyme, Cambridge, MA) in Europe [16], [17], [18] or in Japan [19]. Both two hATG preparations showed similar efficacy for SAA patients with response rates of 60–70% and hATG was herein considered the best choice for SAA. In contrast, a rabbit ATG (rATG) preparation, named as Thymoglobulin® (Genzyme, Sanofi Company, Cambridge, MA) has shown substantial activity as salvage therapy for refractory or relapsed SAA [20], [21] and relatively limited efficacy data were available for Thymoglobulin® as initial therapy in SAA patients [22], [23], [24].

Our previous data of equine ALG (eALG, Lymphoglobulin, Merieux, Lyon, France) plus CSA in SAA patients showed a satisfactory response (78.7%), and a lower response (53.2%) by another rATG preparation (ATG-Fresenius) according to the standard protocol [25]. The amazing differences between ATG-Fresenius and eALG pushed our effort to modify the protocol to strengthen the advantage of rabbit ATG preparations. Thymoglobulin® (Genzyme, Sanofi Company, Cambridge, MA) was the only available agent from the beginning of 2004 in China. Until June 2011, 175 consecutive SAA patients were administrated with rATG in our center, including 51 cases with the standard protocol (3.55 mg/kg per day for 5 days) and 124 patients with an optimized protocol (1.97 mg/kg per day for 9 days). Here, we retrospectively analyzed the data and found that patients received the optimized protocol, along with a moderate and extended immunosuppresion, had a better response and survival.

## Design and Methods

### Patients

We reviewed the records of all newly diagnosed patients with SAA consecutively referred to Blood Disease Hospital of CAMS & PUMC between January 2004 and June 2011 and treated with IST. Patients with a past history of myelodysplastic syndrome (MDS), overt paroxysmal nocturnal hemoglobinuria (PNH), congenital AA and children younger than 5 years old were not included. From January 2004 to June 2006, patients received standard protocol of rATG administration (Standard rATG); thereafter we refined the standard protocol as the reasons mentioned above, and from July 2006 to June 2011, patients received an optimized protocol of rATG (Optimized rATG). The IST protocols described in this study were approved by Ethics Committee, Institute of Hematology & Blood Disease Hospital, CAMS & PUMC. All patients or their legal guardians signed written informed consent before IST.

### Immunosuppressive therapy protocol

Standard rATG (Thymoglobulin®, Genzyme, Sanofi Company, Cambridge, MA) was administered at a dose of 3.55 mg/kg per day for 5 days for SAA patients enrolled from January 2004 to June 2006. With the same total dose of rATG (17.8 mg/kg), Optimized rATG was administered at a dose of 1.97 mg/kg per day for 9 days for SAA patients enrolled from July 2006 to June 2011. To prevent or ameliorate serum sickness, methylprednisolone 1.5 mg/kg per day was started on the first day of rATG for 14 days, and then tapering down and stopping on day 30. All patients received CSA at a dose of 3 mg/kg per day in adults or 5 mg/kg/d in children, which was adjusted according to serum levels. Short courses of recombinant human granulocyte colony-stimulating factor (rhuG-CSF) were administered as clinically indicated, usually for severe systemic infections in both groups. All patients were cared for in rooms with laminar air flow (LAF) 1 week before rATG and continued until an appropriate neutrophil response as ANC>1×10^9^/L. No patients received prophylactic antibiotic support. Red blood cells (RBC) were transfused when hemoglobulin level was less than 70 g/L, and platelets were transfused when platelet count was less than 10×10^9^/L, or less than 20×10^9^/L in the presence of bleeding and/or fever.

### Definitions

SAA was defined as bone marrow cellularity of less than 30% and satisfying two of the three following peripheral blood count criteria: (i) absolute neutrophil count (ANC)<500/µL; (ii) absolute reticulocyte count <60×10^3^/µL; or (iii) platelet count <20×10^3^/µL [15]. Patients were classified as VSAA if they met the criteria for SAA and had an ANC less than 200/µL [26]. Hematological response was defined as no longer meeting criteria for SAA without transfusion or G-CSF. Complete response (CR) was defined as satisfaction of all three peripheral blood count criteria: (i) hemoglobin ≥11 g/dL; (ii) ANC≥1500/µL; (iii) platelet count ≥80×10^3^/µL (the normal platelet count range is of 100–300×10^3^/µL according to the long-term established clinical standard in China instead of 150–350×10^3^/µL in Europe and North America). Partial response (PR) was defined as blood counts no longer meeting criteria for SAA without transfusions and G-CSF, and no response (NR) was classified as still meeting criteria for SAA or continuous transfusion dependency. Relapse was defined as a responder who met criteria for SAA again after achieving response and keeping stable blood counts for at least 3 months. Evolution to MDS was defined as the presence of characteristic dysplastic abnormalities in at least two bone marrow lineages with or without abnormal karyotype. The diagnosis of overt PNH was established by more than two times of positive Ham test or two series glycosylphosphatidyl-anchored protein (GPI-AP)-deficient clone more than 20%, with clinical and laboratory evidence of hemolysis. PNH clone was determined by flow cytometric analysis, detection of more than 5% GPI-AP-deficient clone was considered positive. Early mortality was defined as death occurring within 3 months after initial of rATG.

### Statistical analysis

Summary statistics, such as means, medians and percentiles were used to describe the baseline characteristics of the patients. Categorical data were summarized as frequency counts and proportions. To compare the differences between recipients of Standard and Optimized rATG, the Chi-square test or Fisher exact test was used for categorical variables, and the Mann Whitney-U test for continuous variables. Overall survival (OS) was determined from the initial of rATG to death or last follow-up. Survival curves were calculated using the Kaplan-Meier method and compared using the log-rank test. The Cox regression was used to assess the prognostic factors for survival in multivariate analysis. All significance tests were 2-sided. All the analyses were performed using statistical package SPSS 16.0 (SPSS, Inc., Chicago, IL).

## Results

### Patients' characteristics

A total of 175 SAA patients were treated with IST, including 51 recipients of Standard and 124 of Optimized rATG, respectively. There were no significant differences with regard to demographic or clinical characteristics between recipients of Standard and Optimized rATG (Table 1). The median follow-up was 31.5 months (range, 6.0 to 95.0 months) for all patients, 72 months (range, 6 to 95.0 months) and 30 months (range, 6 to 65.0 months) for recipients of Standard and Optimized rATG, respectively.

**Table 1:** Characteristics of the cohort of patients.

Baseline Characteristics	All patients	Standard rATG	Optimized rATG	*P* value
	(n = 175)	(n = 51)	(n = 124)	
Gender				
Male, No. (%)	104(59.4)	33(64.7)	71(57.3)	
Female, No. (%)	71(40.6)	18(35.3)	53(42.7)	0.36
Median age (years, range)	19(5–62)	18(5–62)	20(5–59)	0.44
Age groups				
<20 yr, No. (%)	100(57.1)	31(60.8)	69(55.6)	
20∼40 yr, No. (%)	53(30.3)	16(31.4)	37(29.8)	
>40 yr, No. (%)	22(12.6)	4(7.8)	18(14.5)	0.50
Etiology of SAA				
Idiopathic, No. (%)	166(94.9)	48(94.1)	118(95.2)	
Hepatitis, No. (%)	9(5.1)	3(5.9)	6(4.8)	0.72
Severity of disease, No.(%)				
SAA	83(47.4)	23(45.1%)	60(48.4)	
VSAA	92(52.6)	28(54.9)	64(51.6)	0.69
Cell counts (median, range)				
ANC(×10^9^/L)	0.19(0–2.59)	0.11(0–2.42)	0.19(0–2.59)	0.34
RET(×10^9^/L)	7.1(0.4–63.2)	6.8(0.8–49.8)	7.2(0.4–63.2)	0.82
PLT(×10^9^/L)	7(0–17)	8(0–14)	6(1–17)	0.58
ALC(×10^9^/L)	1.32(0.21–3.91)	1.29(0.34–3.90)s	1.34(0.21–3.91)	0.71
Interval from diagnosis to rATG (median, range)	23(4–1050)	21(4–486)	24(4–1050)	0.34

*rATG, rabbit antithymocyte globulin; VSAA, very severe aplastic anemia; SAA, severe aplastic anemia; ANC, absolute neutrophil count; ALC, absolute lymphocyte count.*

### Hematological response

The hematologic response rates of all 175 patients were 36.6% at 3 months and 56.0% at 6 months, respectively (Table 2). Outcomes of 51 recipients of Standard rATG were amazingly poor, only 13 (25.5%, 1CR+12PR) and 21 (41.2%, 4CR+17PR) patients achieved hematologic responses at 3 months and 6 months, respectively. Of the 124 patients received Optimized rATG, the hematologic response rates were 41.1% (51/124) and 62.1% (77/124) at 3 and 6 months, respectively; CRs were observed in 9 (7.2%) and 30 (24.2%) patients at 3 and 6 months, respectively. The difference of the response rates at 6 months (*P* = 0.01) between the two groups was significant while it was not at 3 months (*P* = 0.14).

**Table 2:** Hematological response to Standard and Optimized rATG protocol.

Time	All patients	Standard rATG	Optimized rATG	*P* value*
	(n = 175)	(n = 51)	(n = 124)	
At 3 months				
Response, No. (%)	64(36.6)	13(25.5)	51(41.1)	
Complete response	10(5.7)	1(2.0)	9(7.2)	
Partial response	54(30.9)	12(23.5)	42(33.9)	
No Response, No. (%)	111(63.4)	38(74.5)	73(58.9)	0.14
At 6 months				
Response, No. (%)	98(56.0)	21(41.2)	77(62.1)	
Complete response	34(19.4)	4(7.9)	30(24.2)	
Partial response	64(36.6)	17(33.3)	47(37.9)	
No Response, No. (%)	77(44.0)	30(58.8)	47(37.9)	0.01

* *Difference between Standard and Optimized rATG protocol.*

**Figure 1:**
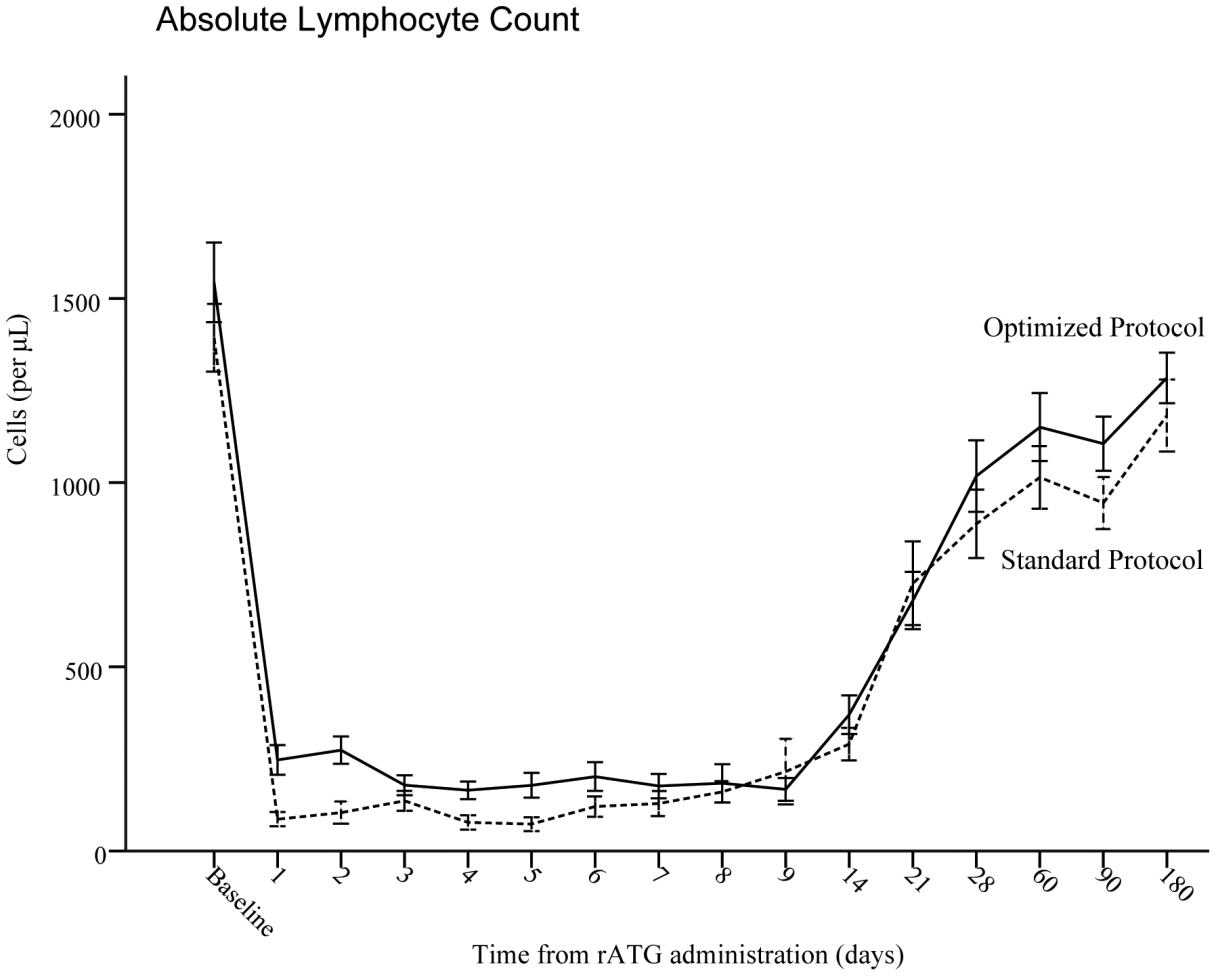
Absolute lymphocyte count (mean±SE) following rATG administration. The degree and duration of lymphopenia were similar between Standard rATG (dotted line) and Optimized rATG (solid line) 9 days and 6 months following rATG administration.

We further evaluated the different potential of lymphocytes depletion between recipients of Standard and Optimized rATG. Rapid lymphodepletion occurred following both protocols, but absolute lymphocyte count was significantly lower in the recipients of Standard rATG in the first seven days of rATG administered excepted on day 8 and 9 (*P*<.001, Figure 1).

### Adverse events and early mortality

Serious adverse events, including early mortality episodes were summarized in Table 3. The incidence of serum sickness in Standard protocol was 68.6% compared with 69.4% in Optimized protocol (*P* = 0.9). But the incidences of infection in 3 months and early mortality in the patients received Standard rATG were significantly higher than those in the Optimized rATG (Table 3). 28 of 51 (54.9%) recipients of Standard rATG experienced infection complications in 3 months; in contrast, the incidence of infection was 37.9% (*P* = 0.04) in recipients of Optimized rATG. The incidence of sepsis within 3 months (16/51, 31.4%) in patients received Standard rATG was significantly higher than recipients of Optimized rATG (7/124, 5.6%; *P*<0.001), mostly due to gram-negative bacterial septicemia. Furthermore, 10 of 51 (19.6%) recipients of Standard rATG died within 3 months, including 8 cases of infection-related death and 2 intracranial hemorrhage. Only 1 (0.8%) infection-related death occurred in the recipients of Optimized rATG (*P*<.001).

**Table 3:** Adverse events in 3 months after IST.

Adverse Events	Standard rATG	Optimized rATG (n = 124)	*P* value*
	(n = 51)	(n = 124)	
Serum sickness, No. (%)	35(68.6)	86(69.4)	0.90
Infection, No. (%)	28(54.9)	47(37.9)	0.04
Early mortality, No. (%)	10(19.6)	1(0.8)	<0.001

* *Difference between Standard and Optimized rATG protocol.*

### Survival

The 5-year OS of all 175 patients was 66.6%±5.2%. Of the 23 deaths in recipients of Standard rATG, 16 resulted from infections and 3 hemorrhagic episodes, 4 occurred after MDS/leukemia transformation. Of the 16 deaths in recipients of Optimized rATG, 13 resulted from infections and 2 hemorrhagic episodes, 1 occurred after MDS transformation.

**Figure 2:**
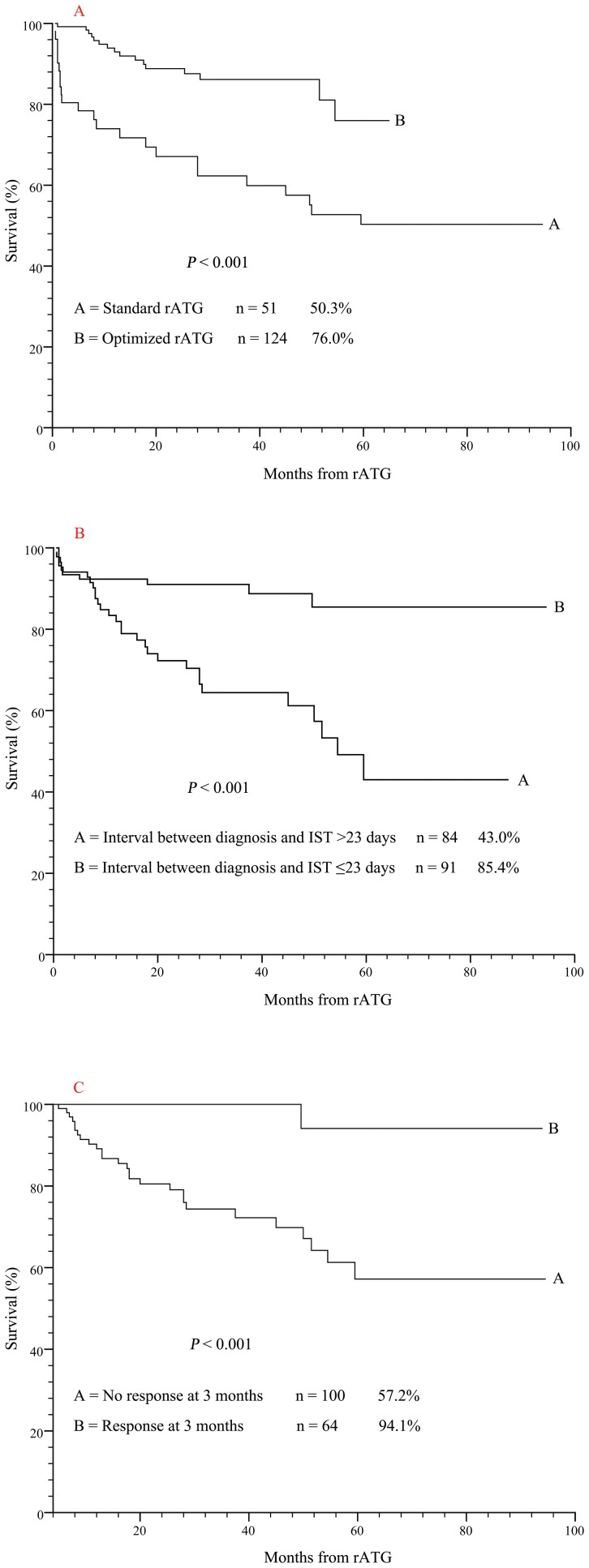
Kaplan-Meier curve of overall survival. A, The 5-year OS of patients received Optimized rATG was superior to that of patients received Standard rATG (*P*<.001). B, Longer interval between diagnosis and IST (>23 days) predicted a worse 5-year OS (*P*<.001). C, The patients with response at 3 months had significantly higher 5-year OS (*P*<.001).

The inferiority of OS at 5 years was 50.3%±7.5% in recipients of Standard rATG compared with 76.0%±7.4% in recipients of Optimized rATG (*P*<0.001), estimated by the Kaplan-Meier method (Figure 2A). Longer interval between diagnosis and IST (>23 days) also predicted a lower 5-year OS (43.0%±9.2% versus 85.4%±4.8%, *P*<0.001, Figure 2B). Post-treatment status was strongly associated with long-term survival. The patients with response at 3 months had significantly higher 5-year OS (94.1%±5.7%), as compared with the patients with no response (57.2%±7.4%, *P*<0.001, Figure 2C). In multivariate analysis, Optimized rATG protocol [RR = 2.21, 95% confidence interval (95% CI, 1.04 to 4.72), *P* = 0.04], response at 3 months [RR = 10.31, (95% CI, 1.35 to 72.92), *P* = 0.03] and shorter interval between diagnosis and IST (<23 days) [RR = 5.35, (95% CI, 1.81 to 15.80), *P* = 0.002] were identified as independent favorable predictors of survival (Figure 2). Sex, etiology, age, ALC, ARC and PLT were not correlated with survival.

**Figure 3:**
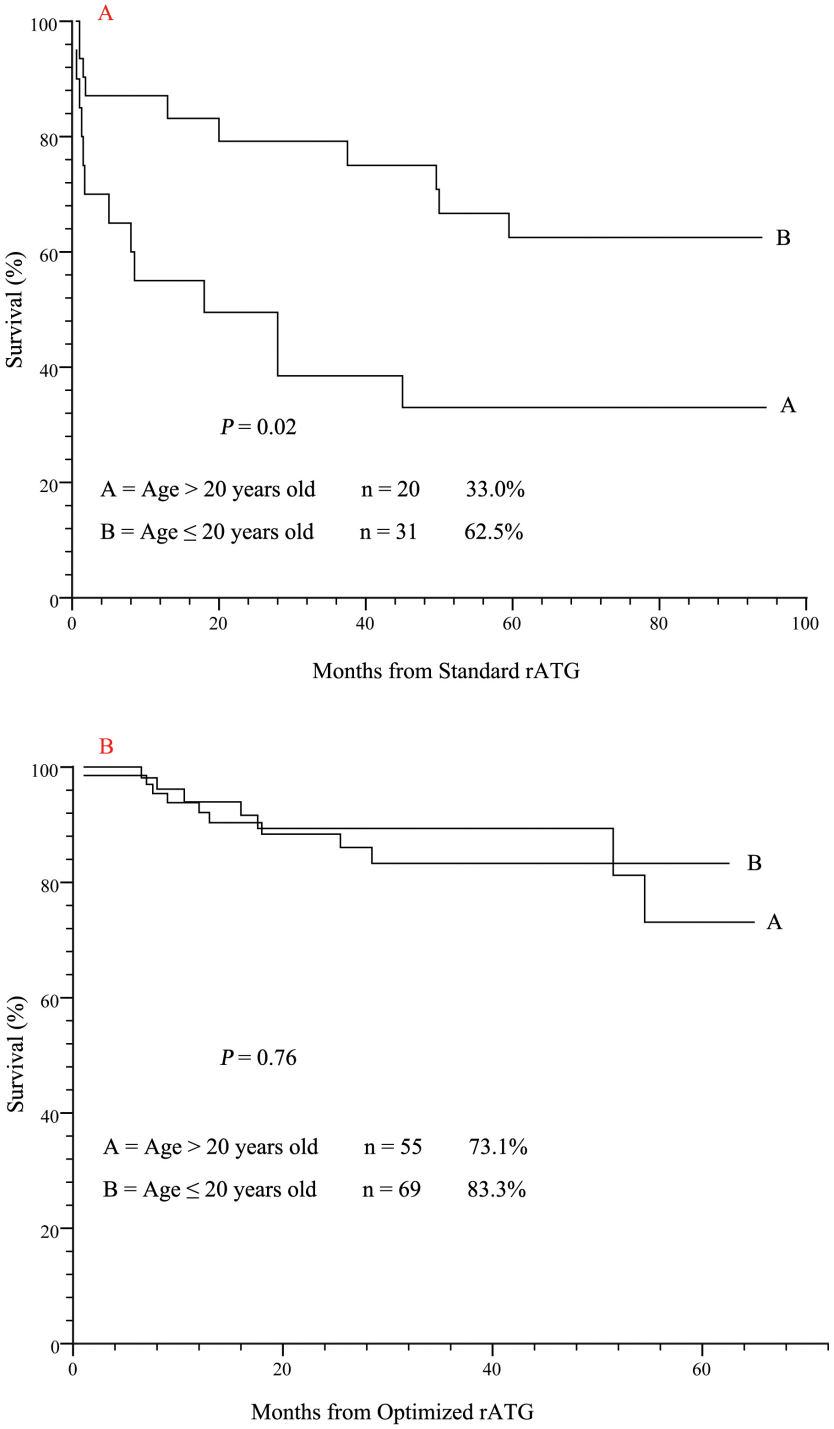
Different OS between younger (≦20 years) and older (>20 years) patients stratified by Standard rATG and Opitimized rATG. A, Following Standard rATG, older patients had a worse OS than younger ones (*P* = 0.02). B, Following Optimized rATG, older and younger patients had a similar OS.

However, when patients were stratified for their treatment protocol, there was difference between patients younger than 20 years old and those older: the 5-year OS in younger and older patients was 62.5%±9.6% versus 33%±10.8% in Standard rATG (*P* = 0.02, Figure 3A), and 83.3%±5.3% versus 73.1%±11.0% in Optimized rATG (*P* = 0.76, Figure 3B). There was also a trend of worse 5-year OS in VSAA patients received Standard rATG (41.0%±10.0% versus 61.4%±10.8%, *P* = 0.11), but this trend disappeared in those received Optimized rATG (74.0%±9.1% versus 77.7%±12.6%, *P* = 0.19).

### Clonal evolution and relapse

In this cohort, 10/175 patients (5.7%) evolved to clonal disorders, including 6/51 (11.8%) recipients of Standard rATG and 4/124 (3.2%) of Optimized rATG (*P* = 0.07). Of the 10 patients, 4 patients progressed to overt PNH, 5 MDS and 1 acute myeloid leukemia (AML). Cytogenetic abnormalities predominantly involved chromosome 7 occurred in 4 cases with MDS and 1 with AML.

A total of 12 (6.9%) patients relapsed at a median interval of 17.5 months (range, 9–47 months) after initial response, including 5 recipients of Standard rATG and 7 of Optimized rATG (*P* = 0.34). 5 relapsed patients acquired response again with the administration of CSA.

## Discussion

rATG has been widely used in conditioning regimens for solid organ and hematopoietic stem cell transplant, limited data were available about rATG as a primary treatment in SAA, rATG (Thymoglobulin®) as the only available agent from the beginning of 2004 in China was administered at a dose of 3.5 mg per kilogram per day for 5 days initially, as previously reported by Di Bona [20], its impressive disadvantages of high incidences of infection (54.9%) in 3 months and early mortality (19.6%) pushed our effort to refine the protocol of rATG. Data from a non-human primate model suggested that T-cell depletion by rATG was dose-dependent [27], absolute lymphocyte count was significantly lower in renal transplant patients conditioned with short-course induction immunosupression with rATG in a prospective, nonrandomized trial [28]; this interesting phenomenon also was seen in our study (Figure 1). There was also a trend towards increases in CMV disease, herpes zoster virus infection and death from infection in the highest dosage (10 mg/kg) group in a retrospective study which compared the efficacy of four different total doses of rATG [29]. From these experiences of rATG in transplant, we supposed that the more potent immunosuppression of rATG was a “double-edged sword” and the “standard” protocol of short course (5 days) of rATG could result in life-threatening immunosuppression due to sledge-hammer destruction of blood cells. We herein refined the “standard” protocol by prolonging the course of rATG administration (1.97 mg/kg/d for 9 days) based on the equality of total dose. The core principle of modification of standard rATG protocol was smooth administration of rATG in order to achieve moderate and sustainable immunosuppressive efficacy. We supposed that different extent of T cell suppression between the two protocols could result in different outcomes. Scheinberg et al [24] speculated that rATG had more potent depletion of CD4+ T cells, the latter had other positive effects on hematopoiesis and may be important for hematologic recovery. More prolonged lymphopenia after the use of rATG might impair marrow recovery, because stimulatory cytokines derived from T cells were depleted. It was still unclear whether further intensification of immunosuppression would definitely yield superior outcomes among patients with SAA [11]. As seen in our data (Figure 1), we found that Standard protocol of rATG, as compared with Optimized protocol, indeed had more potent T depletion in the first seven days and the impact kept on going in 3–6 months. So the T cell suppression by Optimized protocol of rATG was moderate, which could lead to little potent lymphocytotoxic effects and more immunostimulatory potential. That might be a key point to the better response obtained from Optimized protocol of rATG.

Recently, a large randomized trial conducted by NIH compared the efficacies of hATG (ATGAM®) and rATG (Thymoglobulin®), both in combination with CSA as front-line therapy for SAA patients, revealing a disappointed response (37%) and survival (70%) in recipients of “standard” rATG compared with hATG [24]. Our data also demonstrated the same disappointed response (41.2%) to Standard rATG at 6 months, worse than our data of ATG-Fresenius (53.2%), but very similar with the data obtained from Cleveland Clinic group (45.0%) [23] and EBMT SAA Working Party (39.0%) [30]. The survival (2-year OS 64.7%) of patients received Standard rATG was similar with the result from NIH (3-year OS 70% ).

It was noted of an obviously higher incidence of infections (54.9%) and early mortality (19.6%) in recipients of Standard rATG compared with those of Optimized rATG (37.9% and 0.8%, respectively). These differences were unlikely to be explained by the variabilities of supportive care and clinical team. Because the same high incidences of infection in the days of rATG administered (27.5% versus 12.1%, *P* = 0.01), also as the reasons mentioned above and more potent immunosuppressive effect than hATG in acute renal-allograft rejection [31], [32], we herein hypothesized that infections was related to the treatment itself. These serious adverse events were also seen in a prospective study from EBMT (the incidence of infections was 63.0% and high infections-related death) [30] and a retrospective analysis conducted in Brazil (early mortality was 24.1% in the rATG group but 11.9% in the hATG group) [22].

The most impressed result of 124 patients received Optimized rATG was of significantly higher response (62.1%) at 6 months when compared with that of the counterpart, this data was approximate to the response rates of hATG in the previous clinical observations. Furthermore, it partly demonstrated the prolonged course of optimized protocol did not result in less immunosuppression. In the present study, univariate analyses showed that pretreatment neutrophil count was the predictor of survival, and the difference was more obvious in recipients of Standard rATG. However, this predictive significance was disappeared when patients with early mortality were excluded (data not shown). Optimized rATG protocol, response at 3 months and shorter interval between diagnosis and IST predicted the favorable survival by multivariable analyses.

Our data was different from the previous reports and showed an expectedly low probability of clonal evolutions in SAA (5.7%). The discrepancy was at least partly due to different patient populations, different ATG preparations and stringency of measurements for diagnosis of SAA. Low incidence of relapse maybe due to the slow tapering of CSA, which was seen in the previous study [33]. We noticed the data of high incidences of clonal evolution (21.0%) and relapse (28.0%) in hATG group, whereas 14.0% clonal evolution and 11.0% relapse in rATG group in the NIH trial [24], data from Cleveland Clinic group and Brazil disclosed the similar results [22], [23]. Overall, it seemed that rATG reserved the advantages of lower incidence of late clonal evolution and relapse over hATG, though larger series of trials were warranted to confirm this issue.

In summary, the outcomes of Optimized over Standard rATG are in keeping with individual ATG preparation-based immunosuppressive strategy. With the prominent outcomes similar with those of hATG, Optimized instead of Standard rATG in combination with CSA remains efficacious as a first-line immunosuppressive regimen for SAA, which should be strengthened by further prospective study.
